# On absence and abundance: biography as method in archival research

**DOI:** 10.1111/area.12329

**Published:** 2017-03-01

**Authors:** Jake Hodder

**Affiliations:** ^1^ School of Geography University of Nottingham Nottingham NG7 2RD

**Keywords:** archives, absence, abundance, pacifism, biography

## Abstract

Geographical scholarship has rightly problematised the act of archival research, showing how the practice of archiving is not only concerned with how a society collectively remembers, but also forgets. As such, the dominant motif for discussing historical methods in geography has been through the lens of absence: the archive is a space of ‘traces’, ‘fragments’ and ‘ghosts’. In this paper I suggest that the focus on incompleteness and partiality, while true, may also belie what many geographers working in archives find their greatest difficulty: an overwhelming volume of source materials. I reflect on my own research experiences in the pacifist archive to suggest that the growing scale and scope of many collections, along with the taxing research demands of transnational perspectives, pose immediate practical challenges for geographers characterised as much by abundance as by absence. In the second half of the paper, drawing on recent scholarship in history and geography, I argue that the method of biography offers one possible strategy for navigating archival abundance, allowing geographers to tell stories that are wider, deeper and more revealingly complex within the existing time and financial constraints of humanities research.

## Introduction

Introducing students to archival research in *Key methods in geography*, Iain Black tellingly quotes the medieval historian Lynn White Jr who writes, ‘history does not exist; all that exists is debris – scattered, mutilated, very fragmentary – left by vanished ages’ (2010, 467). In grappling with this ‘debris’, geographers have rightly problematised the act of archival work: that the past is wholly, or even largely, recoverable; or that it can be ordered and catalogued in a broadly objective way. If archives are spaces of a society's collective ‘memory’, so too are they sites of loss, effacement and forgetting, where some voices are silent or silenced. In attending to the incompleteness of archives, metaphors of partiality and absence have become the prevailing motif through which to frame discussions of historical research in geography; the archive is a space of ‘traces’ (McGeachan 2016; Ogborn 2005), ‘fragments’ (Till 2001) and ‘ghosts’ (Edensor 2005; Mills 2012, 2013).

Reflecting on my own research experiences in the pacifist archive, however, I explore how the growing scope and scale of many collections, along with the taxing research demands of transnational perspectives, pose immediate practical challenges for geographers working in archives characterised as much by abundance as by absence. These are warning signs of the coming times, as historians, geographers and archivists grapple with the enormous technological and methodological demands of the digital age. If, as Hayden Lorimer (2009) encourages, we are to reflect more frankly on the act of doing historical research, a key component to this must be greater attentiveness to the difficulties faced by geographers as we confront what Roy Rosenzweig suggested might be ‘a fundamental paradigm shift from a culture of scarcity to a culture of abundance’ (2003, 739). Like Rosenzweig wrote of historians, many geographers will similarly recoil at the thought of having to write with even more source material. All the while the subject of abundance (and practical strategies for managing it) rarely features in methods’ texts – often seen as a peculiarly ‘technical issue’ resigned to specialist archive journals or water‐cooler talk.

In the second half of the paper, I suggest that biography offers one possible strategy for coping with abundance. I do so by making a distinction between biography as subject and biography as method: divided by a focus on the internal motivations of past lives and the external factors that shaped them; between a life as text or context. The latter implies a different kind of biographical work, one that is less concerned with knowing a life *per se* than how those experiences can cast light on the wider social and cultural worlds that a life inhabits. I argue that biography conceived in this way can be used instrumentally, a qualitative ‘sampling’ device, to use David Lambert's (2014) observation, to navigate abundant archive collections without compromising on conceptual ambition. It allows consideration of the widest range of material by restricting the relevant volume of material to tell stories that are wider, deeper and more revealingly complex within the existing time and financial constraints of humanities research.

## Archives and absence: traces, fragments, ghosts

Like many areas of the discipline, historical geographers have a long tradition of reflecting on the nature of their craft. As Richard Schein (2001) noted, from the 1970s onwards discussion of methodology complemented a burgeoning interest in historical geography more generally. This culminated in Alan Baker and Mark Billinge's (1982) major edited volume *Period and place*, which for more than two decades stood as the key methods text in the sub‐discipline. Yet in the intervening years a sustained treatment of the practice of historical geography in either book form or progress report has been largely absent (Gagen *et al*. 2007). So too has it traditionally been absent in historical geography papers and monographs themselves, with discussion of sources rarely equating to more than a sentence or footnote. This has led some to question why historical geographers have been so reluctant to describe the instrumental act of working in archives, and to confront the difficult ethical questions that arise from writing about the dead (Lorimer 2009; Moore 2010). Perhaps archival research is seen as instinctively straightforward, Lorimer suggested, or else its individual nature is not conducive to group methodological reflection. While these criticisms continue to hold weight, more recently there has been a growing effort in historical geography scholarship (Bailey *et al*. 2009), methods’ texts (Craggs 2016; Ogborn 2010, 2011) and emerging fields like participatory historical geography (DeLyser 2014) to challenge such silences.

Geographers have been active, however, in exploring the conceptual and political dimensions of archives, commonly bracketed with the broader ‘archival turn’ in the arts and humanities since the early 1990s. Associated with post‐colonial scholarship and interventions by Michel Foucault (2002) and later Jacques Derrida (1996), this turn situated the emergence of archives within the context of the historical development of systems of governance, power and control. In rethinking ‘the materiality and imaginary of collections and what kinds of truth‐claims lie in documentation’ (Stoler 2002, 94), the archive was repositioned as a subject of study in its own right, so that the problem of reading archives became a central concern for those working in them.

Drawing on such approaches, geographers have critically explored the political construction of archives ranging from imperial (Craggs 2008) and disciplinary (Withers 2002) records to personal collections (Ashmore *et al*. 2012) and photography (Rose 2000). In so doing, they have shown how the act of archiving is invariably one of both preservation and discardment, which, in revealing certain aspects of the past, conceal others. Archiving practices determine how value is unevenly ascribed, marking some connections as familiar, while obscuring other subjects or groupings that may cut across archive materials. These silences are not an obstacle to good research, however, but can be its object (Lorimer and Philo 2009). A serious engagement with absence can politicise archiving practices; a kind of ‘remembering [which] implies an ethics about confronting and understanding otherness’ (Edensor 2005, 847). As Dwyer and Davies (2010) documented, there is a growing scholarship in historical geography that deals with marginalia – the unarchived or uncatalogued – deploying a range of innovative techniques to animate such ‘collections’, with Caitlin DeSilvey's (2007) work on a decaying homestead in Montana a prime example. Geographers’ role in historical research is not simply to find documents, therefore, but to situate them within the context of the archive as a site of competing interests. Yet this focus on partiality, incompleteness and absence in geographical literatures on historical methods may belie what many working in archives find their greatest challenge: the disorientating, overwhelming, bewildering volume of presence.

## Archives and abundance: traces by the thousand

Archives are spaces configured by both scarcity and abundance. As much as they are haunted by ghosts and scattered with fragments and traces, so too are these traces measured by the thousand; by the ‘kilometres they span [across] linear meters of shelves’, in ways that are both ‘unsettling and colossal’ (Farge and Davis 2013, 4–5). McGeachan *et al*. offered a familiar description of historical research:

although the archive does not contain *everything*, it can amass rather a lot. Diaries, manuscripts, letters, maps, photographs, ledgers, journals, committee minutes, memos, films and objects to name but a few … For the researcher, this volume of ‘stuff’, that the archive possesses, can be tiresome in nature. There are duplicates and drafts, which are edited and re‐edited, memos which circulate, resurfacing in folder after folder. (2012, 171; original emphasis)

In my own work exploring the historical geographies of radical pacifism in the USA during and after the Second World War, this description rings especially true. Radical pacifism had no distinct organisational form for example, but various activists who worked in a range of peace and civil rights organisations were all orientated towards its basic approach. These activists included the leadership of two of America's largest pacifist groups – the Fellowship of Reconciliation and the War Resisters’ League – which stood alongside any number of more limited campaign groups that, though nominally independent, overlapped in scope, staff and membership – groups like the Congress of Racial Equality or the Committee for Nonviolent Action. Yet their influence reflected an adept ability to contribute to other movements that were not overtly pacifist in constitution in a period in which American power was being consolidated globally. Not only did they play an important role in political events at home, like the early civil rights movement, but many same activists found themselves half‐way around the world protesting in Ghana, Northern Rhodesia or South Africa. This brought the project into contact with more organisations – each with their own large collections, like the American Committee on Africa or American Friends Service Committee – as well as with the vast personal papers of dozens of leading activists.

If radical pacifists operated in and between various movements without any one of them constituting their core focus, similarly that story is collated in and between these movements’ disparate archives. Given the importance of this political flexibility in explaining their influence, narrowing the research to one collection or organisation risked losing the very essence of what gave the movement and its ideas strength. One of the principal challenges therefore was how to use a wide enough range of materials to keep intact the ambition, scope and spirit of radical pacifists, while writing substantively original research, grounded in in‐depth analysis of primary source material.

Discussion of historical methods in geography offers little, however, by way of practical guidance to answer such a question. This is remarkable for two reasons: the shifting technologies of archiving and the recent demands of transnational approaches. First, new technologies have increased our ability to produce and archive large volumes of material. Without the physical constraints of shelving and filing cabinets, the digital era heralds the possibility of archives that are virtually unrestricted in their possible scale, as well as raises a number of technical, political and legal questions. There is an inherent fragility in digital sources. For example, formats quickly become unreadable; information is easily altered, duplicated or lacks the marks of its origins; and it's often unclear who owns it and therefore who has the responsibility to preserve it (see Rosenzweig 2003). Abundance is not the opposite of absence, therefore, but compounds it; we occupy a peculiar moment where we ‘need to be thinking simultaneously about how to research, write, and teach in a world of unheard‐of historical abundance *and* how to avoid a future of record scarcity’ (Rosenzweig 2003, 738; original emphasis). Take for example the Library of Congress's historic announcement in 2010 that it had partnered with the social media website Twitter to preserve every tweet ever posted. Six years later, the archive is still not launched (nor are there any plans to do so) and there is no full‐time member of staff working on the project (McGill 2016). Meanwhile, in theory the collection grows by over 6000 items a second.

These issues are not the sole preserve of so‐called ‘born‐digital’ materials, however, nor for future geographers to ponder alone. Abundance is the product of a longer process by which new technologies of production and circulation have allowed archives to expand unabated. The pacifist archive above, for example, bears the mark of a different (though no less disruptive) technological shift: the explosive growth in the sale of typewriters from the end of the 19th century. In 1880 the number of stenographers and typists recorded in the US census stood at a modest 154. The rapid growth in this number throughout each subsequent decade, rising to 811 190 by 1930, reflects the broader expansion of office culture at the turn of the century (Davies 1982, 178). This transformation has left an unsettlingly large archive of written material in its wake; unsettling in both its sheer volume and in its absences. While the voices of prominent figures can be heard clearly through thick files of correspondence, there is scant acknowledgement to how local people responded to (or participated in) events being undertaken in their own town, city or region (see Hodder 2015). While some voices remain unrecorded, abundance risks drowning out those perspectives that are faintly heard; they are all but lost among a dense administrative circuitry of hundreds of thousands of pages of reports, minutes and press releases.

The second reason that abundance should concern us relates to the growth in transnational approaches to historical research. As Matthew Guterl (2013) has written, the move to transnational history has required stories that are more geographically ambitious across historical periodisations that remain largely unaltered (e.g. Interwar, Post‐war, Cold War); bending space but not time. The outcome is research projects that are more costly, time‐consuming and archive intensive, while funding for humanities research remains tightly constrained. As such, recent research projects show how geographers have developed a range of strategies for managing the archival abundance that such transnational approaches demand. These include the Leverhulme‐funded ‘Snapshots of empire: imperial governance everywhere and all at once’ (University of Sussex, PI: Alan Lester) or the AHRC‐funded ‘Conferencing the international: a cultural and historical geography of the origins of internationalism, 1919–39’ (University of Nottingham, PI: Stephen Legg). These projects use the analytical lenses of the ‘snapshot’ and the ‘conference’ to question how transnational concepts like imperial governance or internationalism ‘touched ground’. While such approaches are theoretically driven, these lenses also provide important practical direction to accessing and processing archive materials – something that, given the nature of the topics, could spread indefinitely and indefinably. In the next section of this paper I suggest that as geographers grapple with abundance, biography offers another way of managing the tasks of shifting, sorting and scoping that have become increasingly central to historical research in geography.

## Biography, ‘like a levee’

Writing biography involves mediating between two competing questions. On one hand is ‘what can we really know of a life?’, and on the other, ‘what can a life reveal to us about something else?’ The relative weighting of these foci distinguishes two different types of biographical scholarship: one is concerned with biography as subject, the other as method. The former question, for example, demands critical reflection on the incomplete nature of the sources one leaves behind. In the case of those most disadvantaged or dispossessed (e.g. labourers, slaves, the subaltern), what we can really know of a life can at times be very, very little, and even with a full historical record a subject's sense of selfhood is rarely if ever constituted in a singular, stable or unified way – what James Clifford called the ‘myth of personal coherence’ (1978, 44). The latter question, however, is less concerned with the internal components of subjectivity than the external factors that shaped it. As the historian Lois Banner writes,

A life deeply lived, like any complex historical narrative, moves across space, time, and areas of human involvement both capriciously and predictably, validating certain accepted historical constructions while challenging others. (2009, 582)

In actuality, good biography often seeks to engage with both of these aspects of a life. In the case of geography, that involves ‘analysing the relationship between individuals’ continually reconstituted subjectivity, the places in which they dwell, and the spaces through which they move’ (Lester 2012, 1470). In light of calls within the discipline to write ‘life geographies’ (Daniels and Nash 2004, 450) and to ‘cast biography in geographical terms’ (Thomas 2004, 498), historical geographers have recently published a diverse array of biographical scholarship, thoughtfully documented in Cheryl McGeachan's (2016) recent progress report. Much of this has developed in novel and experimental ways, ranging from more‐than‐human geographies (Forsyth 2016) and medical histories (Moore 2013) to the role of local lives in the production of geographical knowledge (Matless and Cameron 2007).

Common among these approaches is the question of what difference thinking geographically makes to writing about past lives? ‘Instead of the remorselessly sequential narrative which characterises biographical accounts,’ David Livingstone has written, ‘greater sensitivity to the *spaces of a life* could open up new and revealing ways of taking the *measure of a life*’ (2003, 183, original emphasis). In fact, the recent willingness of geographers and historians (e.g. Colley 2008; Hall 2002; Rothschild 2011) to do biography differently marks a certain sense of renewal for an approach that, as an *American Historical Review* roundtable testifies, has long been disparagingly viewed as ‘the profession's unloved stepchild, occasionally but grudgingly let in the door, more often shut outside with the riffraff’ (Nasaw 2009, 573). Despite a shared commitment to referencing and verification, biography has long been presumed to lack the rigour of serious historical analysis; an ‘abject form of history … a kind of “history‐lite”’ (Munslow 2003, 2).

If some historians remain cautious of labelling their work as biography, this partly stems from the fact that the dead are often pressed into national service, said to reveal something of the collective history or meaning of a nation, embodied in the likes of the *Dictionary of National Biography* (see Baigent 2004). Deacon *et al*. (2010) ask, however, what happens if we take mobility, rather than the nation, as the frame for biographical scholarship? They suggest that just as easily the ‘transnationalism – the mobility, confusion and sheer messiness – of ordinary lives threatens the stability of national identity and unsettles the framework of national histories’ (2010, 2). Similarly, I want to suggest that if we take abundance rather than scarcity as the methodological frame for biography, then a life can be used ‘strategically, like a levee, to direct a story that might spill sideways into other areas, to direct it forward and more forcefully along the transnational course’ (Guterl 2013, 139) as well as through the archive.

In many senses this is what historical geographers have been doing – using biographical material, or ‘traces’ (Ogborn 2005), as part of a repertoire of methods to navigate the abundance thrown up by transnational approaches. This has the benefit of not only being a cost‐effective strategy for sourcing a large volume and range of empirical material, but it also helps conceptually focus on how the life of an individual is interconnected with the institutions, ideas, networks, places and objects with which they came into contact. As David Lambert (2014) noted, imperial biographies force us to consider the inherent relationality of empire, one that simultaneously shaped and was shaped by those who lived it – not only rethinking the spatiality of biography therefore, but also providing new perspectives on global history. As such, a focus on biographies (Anderson 2012; Hall 2002; Lambert 2013; Legg 2010; Lester 2012; Ogborn 2008) and careering (Craggs and Neate 2016; Lambert and Lester 2006) have been especially useful in re‐examining the histories and geographies of empire. There are of course limitations in foregrounding individual lives, and biography fits some projects less easily than others (e.g. those interested in the non‐human or writing historical geography over the longue durée). This paper is not an attempt to claim preferential treatment for biography, however, but rather to simply suggest that it sits within a broader toolkit of approaches available to historical geographers. Moreover, the flexible ways in which biography is currently being used offers real possibilities for grappling with the growing challenges posed by source abundance, not least in my own research to which I now return.

## ‘A little mixing and matching’

In the final part of this paper, I show how the method of biography allowed the issue of abundance to be managed in my own work tracing the transnational histories of radical pacifism. I drew on the life of the civil rights and peace activist Bayard Rustin (Hodder 2014; Hodder 2016). Rustin was the race relations secretary of the Fellowship of Reconciliation from 1941 to 1953, after which he took leadership of the War Resisters’ League. He embodied radical pacifism's internationalist vision, working alongside civil rights and anti‐colonial leaders in the USA, India, Ghana, Nigeria, Tanzania and Zambia during the 1950s and 1960s alone. In 1956, Rustin joined King at the Montgomery Bus Boycott, and over the following decade played a decisive role in shaping the methods and strategy of the civil rights movement.

Rustin's itinerant life, lived in motion, forced an account of radical pacifism forwards, forced it across oceans and borders, and thereby exposed its transnational dimensions. Such histories are often obscured from view by the ‘instituting imaginary’ (Mbembe 2002) of national archives; an organising logic that disperses transnational stories across multiple sites, marking them as somehow peculiar or odd. By using Rustin's biography to screen a more limited selection of archive material, he allowed the project to retain its broad scope while grounding it in in‐depth forms of textual, visual and discourse analysis that require scrupulous examination of primary sources. Episodes of Rustin's international life were teased out in a series of tightly defined case studies that folded the timeline of his biography; some spanned a matter of days and others several years. This took encouragement from the knowledge that geographical biographies do not need to be ‘penned according to a traditional linear structure – a fixed arc of existence that begins, happens and ends’ (Lorimer 2003, 283). Life geographies, by bending space *and* time, allow greater flexibility to chase the scattered remains of transnational lives through the archive. Capturing these contingent histories and geographies often requires a more flexible kind of scholarship; ‘a little mixing and matching, movement and stretching’ (Schechter 2012, 5).

What was left was neither a plotted history of radical pacifism, nor a biography of Rustin, but a series of loose intersections that revealed the larger aim of the project: to explore the entangled histories of the civil rights movement, African anti‐colonialism and the radical peace movement. Rustin was especially useful in this regard because he was a well‐known figure whose international work had been largely overlooked. As the day‐to‐day shape of his American life had already been dutifully mapped out (e.g. Anderson 1997; D'Emilio 2003), it gave the potential to engage with his biography in a more selective way. His footloose life reflected a deeper nature in his personality that was ill‐suited to a sedentary existence, the

details of administration bored him. He much preferred life as a troubleshooter – an itinerant strategist, tactician and organizer. Running off here and there … [rather] than presiding steadily at an office desk. (Anderson 1997, 4)

Rustin, off ‘here and there’, was used to both contain the story of radical pacifism and force out its transnational dimensions that are often blurred in day‐to‐day administrative records.

This is less traditional biography than what Alice Kessler‐Harris (2009) playfully calls writing ‘anti‐biography’ – instead of using history as background to locate an individual in time, a life moving in and out of view can force historical processes into the foreground. Biography acts as a connective device, allowing one to plot a path through abundant archives without compromising on conceptual or theoretical ambition. While in my own project Rustin's presence loomed large, for example, he never became its focus. Instead, he provided continuity and direction among a larger cast of characters, institutions, places and networks that formed and disbanded at specific times and in response to specific political challenges. As one reviewer wrote of Neil Smith's work on Isaiah Bowman, Rustin similarly was

never completely lost in these discussions, but like a plot‐connective character in a novel or play, he disappears from sight occasionally and at other times stands awkwardly at the edges of the main plot action. (Entrikin 2003, 267–8)

## Conclusion

In this paper I have argued that although geographers have rightly interrogated the archive as a space of absence, so too is it one of abundance. However, practical strategies for coping with the latter are often lost or buried within geographical literatures on historical methods that are overwhelmingly concerned with issues of incompleteness, fragmentation and partiality. I have suggested that this asymmetry is surprising for two reasons. First, because new technologies of production, circulation and storage have led to a rapid growth and expansion of archival repositories, a condition any historian of the 20th century is intimately familiar with, and second, because the recent turn to transnational perspectives and global history has placed enormous demands on researchers to draw from a greater range of archives and materials than was previously the case.

Considering these challenges, I have made a pragmatic case for biography, as a method, to be included in the historical geographers’ toolkit, and reflected on my own experiences researching in the pacifist archive to illustrate this point. As such, biography can help us negotiate what Simon Naylor called ‘the always‐more‐than‐local nature of the world … [histories that are] not just local and particular, but not easily universal and generalizable either’ (2008, 271). It does so by providing the opportunity to practically research the past in a way in which its cracks and slippages are ever‐present. As we confront the methodological and technological demands of researching, writing and teaching in a new age of source richness and plenty, the job of sorting and scoping will become increasingly important and biography offers one framework to organise these in more purposeful and sophisticated ways. Rather than narrowing our research interests, this paper has argued that by charting a course through the abundant archive, biography allows us to tell stories that are bigger, bolder and broader, and to do so in more interesting ways, where the people and places of the past are made ‘more human, more vivid, more intimate, more accessible, more connected to ourselves’ (Schlesigner Jr 2002, xiv).
